# Opposed Interplay between IDH1 Mutations and the WNT/β-Catenin Pathway: Added Information for Glioma Classification

**DOI:** 10.3390/biomedicines9060619

**Published:** 2021-05-30

**Authors:** Alexandre Vallée, Yves Lecarpentier, Jean-Noël Vallée

**Affiliations:** 1 Department of Clinical Research and Innovation, Foch Hospital, 92150 Suresnes, France; 2 Centre de Recherche Clinique, Grand Hôpital de l’Est Francilien (GHEF), 77100 Meaux, France; yves.c.lecarpentier@gmail.com; 3 Centre Hospitalier Universitaire (CHU) Amiens Picardie, Université Picardie Jules Verne (UPJV), 80000 Amiens, France; valleejn@gmail.com; 4 Laboratoire de Mathématiques et Applications (LMA), UMR CNRS 7348, Université de Poitiers, 86000 Poitiers, France

**Keywords:** gliomas, glioblastomas, WNT/β-catenin pathway, IDH1, cancer, hypoxia, normoxia

## Abstract

Gliomas are the main common primary intraparenchymal brain tumor in the central nervous system (CNS), with approximately 7% of the death caused by cancers. In the WHO 2016 classification, molecular dysregulations are part of the definition of particular brain tumor entities for the first time. Nevertheless, the underlying molecular mechanisms remain unclear. Several studies have shown that 75% to 80% of secondary glioblastoma (GBM) showed IDH1 mutations, whereas only 5% of primary GBM have IDH1 mutations. IDH1 mutations lead to better overall survival in gliomas patients. IDH1 mutations are associated with lower stimulation of the HIF-1α a, aerobic glycolysis and angiogenesis. The stimulation of HIF-1α and the process of angiogenesis appears to be activated only when hypoxia occurs in IDH1-mutated gliomas. In contrast, the observed upregulation of the canonical WNT/β-catenin pathway in gliomas is associated with proliferation, invasion, aggressive-ness and angiogenesis.. Molecular pathways of the malignancy process are involved in early stages of WNT/β-catenin pathway-activated-gliomas, and this even under normoxic conditions. IDH1 mutations lead to decreased activity of the WNT/β-catenin pathway and its enzymatic targets. The opposed interplay between IDH1 mutations and the canonical WNT/β-catenin pathway in gliomas could participate in better understanding of the observed evolution of different tumors and could reinforce the glioma classification.

## 1. Introduction

Gliomas are the main common primary intraparenchymal brain tumor in the central nervous system (CNS), with approximately 7% of the death caused by cancers [1,2]. Gliomas are composed by a very heterogeneous group of primary CNS tumors, originally classified according to their microscopic similarity with presumed origin of non-neoplastic glial cells (including astrocytes—astrocytoma; oligodendroglial cells—oligodendroglioma; ‘glioblast’—glioblastoma). Gliomas are traditionally divided into two major categories: ‘diffuse’ gliomas and ‘non-diffuse’ tumors. Diffuse gliomas are characterized by tumor cell migration over large distances into the CNS parenchyma, with possible curative surgical resection. Malignancy grade was defined by the presence or the absence of marked mitotic activity, necrosis and/or florid micro- vascular proliferation. Non-diffuse gliomas are mainly much more circumscribed. Molecular mechanisms can help to categorize glial tumors into different diffuse and non-diffuse glioma entities. The tremendous increase in knowledge of the molecular characteristics of CNS tumors during the last decade has allowed for a paradigm shift. In the update of the 4th edition of the World Health Organization (WHO) classification CNS tumors published in 2016 [3,4], molecular dysregulations are part of the definition of particular brain tumor entities for the first time. Especially, the classification of the main frequent primary neoplasms of the CNS parenchyma itself, the diffuse gliomas, has undergone major restructuring based on the status of a few key molecular aberrations (Supplementary Materials). Nevertheless, the molecular mechanism that gliomas undergo remains unclear and needs to be clarified.

Mutations in enzymes regulating metabolite flux are implicated in gliomas development, as highlighted by the discovery of isocitrate dehydrogenase 1 (IDH1), in more than 70% of diffusely infiltrating WHO grade II and grade III astrocytic and oligodendroglial gliomas, as well as in a small fraction of glioblastomas (GBM), particularly those that develop from low-grade-gliomas (LGG) [3,5,6,7,8].

In parallel, a large number of studies have suggested that WNT signaling is aberrantly activated in GBM and that it promotes GBM growth and invasion [9,10,11,12,13].

However, very few studies have investigated the interplay between IDH1 mutations and WNT signaling in gliomas and thus, the repercussion in gliomas development.

IDH1 mutations have not been reported in pediatric gliomas, suggesting that IDH1 mutations are only restricted to astrocytomas and oligodendrogliomas in young adults [14,15,16]. Gliomas in children are predominantly driven by other mutations (with mutations at K27M or G34V or G34R). The nature of these different mutations, restricted to certain ages, suggest that pediatric and young adult gliomas may have different cells of origin [17]. Thus, this review is focused on the opposed interplay between IDH1 mutations and the canonical WNT/β-catenin in gliomas development in young adults.

## 2. IDH1 Mutations and Glioma

Glioblastomas (GBM) differ at the genetic and epigenetic levels, especially with the distinguishing feature in the identification of mutations in the metabolic enzyme isocitrate dehydrogenase 1 (IDH1). IDH1 mutations are one of the earliest detectable genetic damages in low-grade gliomas [17]. IDH1 were reported in 2008 and the mutation of the arginine at codon 132 is the main frequent type. It was also reported that 80% to 90% of IDH1 mutations in astrocytic and oligodendroglial gliomas have the type R132H (arginine to histidine) [18,19,20]. Numerous studies showed that IDH1 mutations lead to better overall survival in gliomas patients and better response to therapies [21,22,23,24].

It was found that 75% to 80% of secondary GBM showed IDH1 mutations, whereas only 5% of primary GBM had IDH1 mutations [17,25]. Primary GBM were observed to have very low mutations levels in IDH1 (around <3%–5%)) [17,26]. IDH1 mutations had developed through progression from an anaplastic glioma (WHO Grade III), while the majority of secondary GBM with IDH1 mutations had progressed from a WHO Grade II glioma [19]. Moreover, to provide insights about the origin of gliomas, the mutational status of IDH1 serves as a prognostic factor in patients with WHO grade II and III gliomas [27] and GBM [28].

IDH1 is considered as a marker of secondary GBM and those primary GBMs diagnosed with IDH1 mutations may have been secondary gliomas that rapidly progressed to GBM with no early low-grade clinical symptoms experienced by patients [29]. IDH1 mutations occur early in glioma-genesis and occur in a progenitor cell that can give rise to both cell types. IDH1 mutations have not been reported in other CNS tumors including ependymomas, medulloblastomas, meningiomas, and pilocytic astrocytomas and are very rare in spinal gliomas [19,30,31,32]. In gliomas, the frequency of IDH1 mutations in codon 132 increases in the order R132L, R132S, R132G, R132C, to R132H, with R132H constituting more than 90% of all IDH1 mutations [21].

Somatic mutations in IDH1 genes have recently been identified in a large proportion of glial tumors of the CNS [4,21]. IDH1 mutation has been reported to be a strong and independent indicator for good prognosis in gliomas whatever the tumor grade [33].

IDH1 mutation was a positive prognostic marker, because patients with this mutation had significantly better overall survival [5]. Several studies also showed that patients with GBM or diffuse glioma with IDH1 mutations had better overall survival and progression-free survival [7,34,35,36,37,38,39].

Some studies showed that patients with IDH1 mutations versus those with wild-type IDH1 possessed significantly better overall survival times when treated with surgery and radiotherapy [40,41]. IDH1 mutations were associated with a better response to cytotoxic therapy and longer survival in malignant glioma patients [42,43]. In comparison with IDH-mutated LGG, primary GBM (IDH wild-type GBM) and IDH wild-type LGG were characterized by a clinically aggressive behavior with a dismal prognosis [27,44].

## 3. Non-IDH1-Mutated Glioma

Integrated genomic/transcriptome and epigenomic analysis resulted in a gene expression-based molecular classification of GBM into classical, mesenchymal (MES), proneural (PN), and neural subtypes, characterized by aberrations and gene expression of the epidermal growth factor receptor (EGFR), neurofibromatosis Type 1, and platelet-derived growth factor receptor a (PDGFRa). Previous studies showed that responses to aggressive chemotherapy and radiotherapy differed according to subtype [45].

The PN subgroup was enriched for mutations in IDH1, mutation in TP53, and amplifications of PDGFRa, cyclin-dependent kinase 6 (CDK6), CDK4, and receptor for hepatocyte growth factor (Met) [17]. Moreover, this group contained the highest percentage of young patients, likely because of the enrichment of IDH1 mutations, which is associated with younger patient age. The classical subtype is characterized by EGFR amplification and a loss of phosphatase and tensin homolog (PTEN). The classical subtype also harbors the mutant EGFR variant III (EGFRvIII) mutation, which is constitutively active and has an in-frame deletion of exons 2–7. The MES subclass is associated with poor overall survival, contains neurofibromatosis Type 1 mutations, and has a loss of TP53 and CDK inhibitor N2A (CDKN2A). Last, the neural subtype has elevated levels of neural markers such as NEFL but has no unique distinguishing alterations from other classes, although elevated rates of ERBB2 mutation were observed [17].

The genomic characterization of IDH wild-type GBM reveals frequent genetic alterations of key components of the growth factor receptor-PI3K-Akt signaling pathway that activates the mechanistic target of rapamycin (mTOR) signaling [46,47,48]. The EGFR is a major activator of a variety of pathways and physiological responses include proliferation, survival, migration, and tumorigenesis. EGFR is amplified in about 40% of GBM patients, and is often associated with high-grade classical tumors [25]. While it is commonly thought that amplification or mutation of EGFR is an indicator of poor survival, several studies failed to validate this conclusion [49]. Mutations and amplifications of EGFR and loss of chromosome 10 are rarely found in IDH1 mutant tumors [26].

The PI3K pathway is normally activated by the EGFR and other growth factor receptors [50]. EGFR binds PI3K/Akt/STAT and modulates proliferation, differentiation and survival [51,52]. PI3K is activated by EGFR. PI3K converts PIP2 in PIP3 [53,54]. The activity of PI3K is counteracted by PTEN, which converts PIP3 back to PIP2 [55]. PI3K activates Akt signaling by phosphorylation [56]. An increase in Akt signaling inhibits GSK-3β activity and leads to nuclear translocation and stability of β-catenin [53]. PI3K/Akt signaling regulates β-catenin stability, translocation, transcriptional activity and the expression of its downstream genes (such as Cyclin D1 and c-Myc) [54].

Approximately 40% of GBM have mutations in the PTEN protein and around 70% show a loss of heterozygosity at the PTEN locus [57]. The value of PTEN loss as a prognostic marker has not been validated, and is still somewhat controversial [58].

Recent studies identified a set of interlacing molecular mechanisms by which EGFRvIII, a constitutively activating mutant form of EGFR, co-opts c-Myc to reprogram cellular metabolism and drive tumor proliferation. This involves the mTOR complexes 1 and 2 (mTORC1 and mTORC2) [46,59]. Moreover, failure to inhibit mTOR signaling can render GBM cells resistant to PI3K/Akt-targeted therapies by maintaining elevated levels of c-Myc [59].

In EGFR-mutant GBM, which do not usually possess the mutations in IDH or H3 histone family 3A (H3F3A) to potentially change the epigenetics, constitutive PI3K activation could engage the epigenetic machinery through several complementary routes. First, EGFR activation causes the glycolytic enzyme pyruvate kinase isozymes M2 (PKM2) to translocate to the nucleus, where it phosphorylates histone 3 at Thr11, causing dissociation of histone deacetylase 3 (HDAC3), and promotes histone acetylation to regulate transcription of the cancer-promoting genes, including c-Myc and cyclin D1 [60].

In approximately 30% of human gliomas, expression of genes associated with PDGFR signaling and genes involved in oligodendrocyte development (OLIG2, NKX2-2, and PDGF), are observed and are thought to be hallmarks of the PN signature in GBM [9]. Amplification of the α-type PDGFR (PDGFRA) gene is found in 15% of all tumors, mainly in the PN subtype of GBM [45,61] and approximately 40% of tumors harboring gene amplification contain an intragenic deletion in this gene [62]. However, the expression of PDGFRB seems to be limited to proliferating endothelial cells in GBM [63,64]. Similar to EGFR and EGFRvIII, amplification of PDGF and PDGFR seems to promote aggressive glioma growth [65].

## 4. Canonical WNT/β-Catenin Pathway

The WNT name is derived from Wingless drosophila melanogaster and its mouse homolog Int. The WNT/β-catenin pathway is implicated in several mechanisms and controls signaling, including embryogenesis, cell proliferation, migration and polarity, apoptosis, and organogenesis [66]. Nevertheless, during several pathological diseases, the WNT/β-catenin pathway can be altered, to impact mechanisms including inflammation, metabolic, neurological and psychiatric disorders, fibrosis and cancer processes [67].

The WNT pathway belongs to the family of secreted lipid-modified glycoproteins [68]. WNT ligands are produced by neurons and immune cells localized in the CNS [69]. WNT pathway dysfunction could affect numerous neurodegenerative pathologies [70,71,72,73,74]. The WNT pathway has a main stage called the β-catenin/T-cell factor/lymphoid enhancer factor (TCF/LEF). Cytoplasmic accumulation of β-catenin is modulated by the destruction complex AXIN, tumor suppressor adenomatous polyposis coli (APC), and glycogen synthase kinase-3 (GSK-3β). With absence of WNT ligands, the destruction complex has a role in the hyper-phosphorylation of the cytoplasmic β-catenin and leads to its proteasomal destruction. Nevertheless, in their presence, the WNT ligands bind to Frizzled (FZL) and LDL receptor-related protein 5/6 (LRP 5/6) to interrupt the destruction complex and prevents β-catenin degradation into the proteasome. β-catenin translocates to the nucleus to interact with the TCF/LEF; this stimulates WNT target genes [75,76,77].

Glycogen synthase kinase-3β (GSK-3β) is one of the major inhibitors of the WNT/β-catenin pathway [78,79,80,81,82,83]. As an intracellular serine-threonine kinase, GSK-3β is a major negative controller of the WNT signaling [84]. GSK-3β is implicated in the control of numerous kinds of pathophysiological pathways, including cell membrane signaling, cell polarity, and inflammation [85,86,87]. GSK-3β interacts by downregulating the cytoplasmic β-catenin and stabilizing it to enhance its nuclear migration. Inflammation is an age-related mechanism correlated with the activation of GSK-3β pathway and the diminution of the WNT/β-catenin pathway [88].

Recent studies have observed that glaucoma patients presented activation of the GSK-3β pathway and its downregulation may be an interesting therapy target [89,90]. Dysregulation of GSK-3β is implicated in the pathogenesis of numerous pathologies, such as cancer processes [91,92,93]. GSK-3β is a regulator of numerous signaling including inflammation, neuronal polarity or cell membrane signaling [86]. GSK-3β is known to be the major inhibitor of the canonical WNT/β-catenin pathway [82,93,94,95,96,97].

## 5. Canonical WNT/β-Catenin Pathway in Glioma

**Figure 1:**
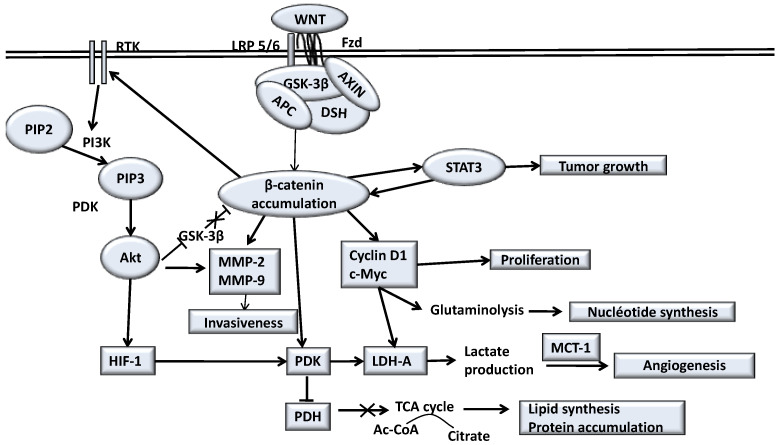
The WNT/β-catenin pathway and its different actions in gliomas processes. When the canonical WNT/β-catenin pathway is stimulated, STAT3 signaling pathway upregulates the expression and transcriptional activity of β-catenin. In cancers STAT3 is a tumor aggressiveness factor. In cancers, the overexpression of EGFR (a receptor tyrosine kinase: RTK) stimulates PI3K/Akt pathway. PI3K/Akt signaling leads to the phosphorylation of GSK-3β that leads to nuclear translocation and stabilization of β-catenin. Similarly, WNT/β-catenin pathway stimulates EGFR in gliomas. Akt signaling increases MMP-2 and MMP-9 activity, which induce invasion of cancer cells. Akt signaling induces HIF-1a, which stimulates PDK1. Overexpression of WNT/β-catenin also stimulates PDK1. PDK1 and c-Myc induce LDH-A, and cytolsolic pyruvate is shunted into lactate through activation of LDH-A. Overexpression of MCT-1 exports lactate to extracellular space. Lactate production stimulates angiogenesis. c-Myc induced glutaminolysis supports mitochondrial integrity and production of aspartate, and results in nucleotide biosynthesis. c-Myc and cyclin D stimulate proliferation of gliomas.

A large number of studies have suggested that WNT signaling is aberrantly activated in GBM and that it promotes GBM growth and invasion via the maintenance of stem cell properties [9,10,11,12,13]. The nuclear accumulation of β-catenin is responsible for the malignant progression and β-catenin protein levels are correlated with malignancy and with the gene expression of cyclin D1 and c-Myc [98,99,100]. The aberrant activation of WNT/β-catenin pathway contributes to gliomas progression [98,99,101,102,103,104,105] (Figure 1).

Recent studies have described the functions of WNT/β-catenin pathway in development and cancer, with particular emphasis on genetic and epigenetic alterations that lead to aberrant WNT pathway activation in GBM [106,107,108,109,110]. Prominent genomic alterations frequently found in GBM include loss-of-function of tumor suppressors in the p53, phosphatase and tensin homolog and neurofibromatosis 1, and hyperactivation of receptor tyrosine kinase (RTK) signaling, including EGFR, PDGF receptor, and the receptor for hepatocyte growth factor (Met) [3].

Epigenetic silencing of negative effectors of WNT pathways can activate WNT signaling and contribute to malignant behavior in GBM. Soluble Frizzled-related proteins (FRPs) are soluble proteins that bind to WNT and interfere with WNT signaling. Dickkopf (DKK) acts as an antagonist of WNT signaling via binding to its co-receptor LRP [111]. Notably, epigenetic silencing of WNT pathway inhibitor genes frequently occurs in gliomas, including promoter hyper-methylation of sFRPs, Dickkopf-1 and Naked (NKD1, NKD2). In GBM, promoter hypermethylation of sFRP1, sFRP2 and NKD2 occurred in more than 40% of primary GBM specimens [112,113].

Epithelial–mesenchymal transition (EMT) is a critical process that enables cancer cells of epithelial origin to metastasize to distal organs. Unsurprisingly, WNT signaling is involved in both tumor invasion and EMT [12]. FZD4, a positive WNT regulator, was identified and shown to be a causative effector for invasive phenotypes of GBM cells [114].

Receptor tyrosine kinases (RTKs) promote GBM survival, proliferation, and invasion. Hyperactivation of RTK signaling because of genomic amplification and/or activating mutations of RTKs occurs in more than 90% of GBMs [45]. Amplifications or somatic mutations in EGFR, PDGF receptor, FGFR, and Met often correlate with GBM subtypes [45].

Activation of EGFR induces downstream mitogenic signaling, such as the mitogen-activated protein kinase, PI3K/Akt, and transducers and activators of transcription (STAT) pathways [115,116]. WNT pathway can induce activation of the Akt pathway [117].

Met has crucial roles in cancer growth, stem cell maintenance, and metastasis [118]. In GBM, expression levels of Met correspond with poor patient survival and malignancy [119]. In addition, analyses of clinical GBM specimens revealed a positive association between Met expression and invasiveness-related genes (matrix metalloproteinases: MMP2 and MMP9) and proto-oncogenes (c-Myc, KRAS, and JUN) [120,121]. Activation of Met signaling can be enabled by the addition of hepatocyte growth factor (HGF)-induced nuclear translocation of β-catenin [122].

## 6. Opposed Interplay between IDH1 Mutations and WNT/β-Catenin Pathway in Glioma

A recent study reported that the IDH1-R132H mutation causes both a less aggressive phenotype and radiosensitization of human malignant glioma cells [123]. Metastasis in cancer is initiated by a process called EMT transition that requires upregulation of β-catenin, the mediator of the canonical WNT pathway [124,125]. IDH1-R132H mutation leads to decreased activity of WNT/β-catenin pathway, that in turn curbs the elevated proliferation and migration observed in invasive gliomas [126]. The expression of mediators, effectors and targets of the canonical WNT pathway, including β-catenin, TCF4 and LEF1 is downregulated in glioma cells overexpressing IDH1-R132H [126]. In addition, negative regulators of WNT/β-catenin pathway, such as DKK1 and APC, are upregulated in these cells. The activity of the endpoint mediator of this pathway, TCF, is significantly downregulated in IDH1-R132H cells [126]. Thus, IDH1-R132H causes a significant reduction in the proliferation, migration and invasiveness of gliomas, accompanied by an increase in apoptotic cell death [127].

Glioma cells overexpressing IDH1-R132H displayed higher chemosensitivity due to increased generation of reactive oxygen species (ROS) and depletion of glutathione, suggesting that they respond better to chemotherapy than IDH1-wt gliomas [127,128].

The PI3K/Akt pathway is upregulated in invasive gliomas in association with the WNT/β-catenin pathway [92,129], and is known to induce tumor progression [130]. IDH1-R132H decreases the activity of the PI3K/Akt pathway in glioma cells [131]. Mutated IDH1 thus blocks PI3K/Akt signaling, a pathway associated with the development of a more aggressive glioma phenotype [132,133].

Recent studies have shown that the presence of IDH1-R132H mutation is associated with a better prognosis in glioma patients in association to the decrease in the expression of the WNT/β-catenin pathway [134]. Yao et al. demonstrated that IDH1 mutation diminished the malignant progression of glioma by causing a less aggressive phenotype of GSCs, which were involved in the WNT/β-catenin pathway [126,134]. Furthermore, IDH1 mutation is associated with the reduction in cell survival, proliferation and invasion of glioma by decreasing the WNT signaling [126,135] (Table 1).

## 7. Oxidative Stress and IDH1 Mutations in Glioma

Previous studies showed that elevated levels in tumor cells of reactive oxygen species (ROS) led to cell cycle arrest, inhibition of proliferation and promotion of apoptosis, thereby decreasing tumor growth [140,141]. An imbalance between the production of ROS and the ability of the cellular antioxidant system to readily detoxify ROS led to oxidative stress [142,143]. Glutathione (GSH) is the most abundant intracellular antioxidant, and is involved in the protection of cells against oxidative damage and in various detoxification mechanisms [144]. Diminution of intracellular GSH levels results in the accumulation of ROS in cells, and elevated levels are associated with apoptosis resistance [145]. Nicotinamide adenine dinucleotide phosphate (NADPH) is an essential cofactor for the biosynthesis of GSH, and the oxidized form of glutathione, GSSG, is reduced to GSH in an NADPH-dependent reaction catalyzed by glutathione reductase [146].

IDH1 catalyzes the oxidative carboxylation of isocitrate to a-ketoglutarate, yielding reduced NADPH [141]. In gliomas, IDH1 mutations decreased the intracellular GSH levels, enhanced the levels of intracellular ROS and inhibited the growth of glioma cells [141]. Overexpression of mutant IDH1 in glioma cells resulted in an increase in intracellular ROS levels, showing that the mutant IDH1 affected growth inhibition by induction of cell apoptosis and inhibition of proliferation [128]. Thus, NADPH levels are diminished in IDH1-R132H gliomas in situ [136].

## 8. Hypoxia in IDH1-Mutated Glioma

Tumor hypoxia results in constitutive upregulation of glycolysis and acidosis, contributing to the tumor resistance of therapeutic agents [147]. The progression of gliomagenesis often occurs in a hypoxic microenvironment that compels the use of anaerobic glycolysis as the primary energy source [148]. Hypoxia stabilizes HIF, a transcription factor, which increases the biological aggressiveness of tumors, promoting glycolysis, cellular proliferation, and angiogenesis [149,150]. Once activated, HIF-1α can regulate the expression of many glycolytic enzymes including glucose transporters and mitochondrial enzymes that are involved in the metabolic adaptation to hypoxia through the conversion of glucose to pyruvate and subsequently to lactate [151]. Hypoxia in solid tumors plays an important role in the propagation of a cascade of molecular pathways in favor of tumor growth [152]. Hypoxia-induced HIFs stimulate angiogenesis and increase malignancy, metastasis, and resistance to therapy [153]. HIF-1α is one of the key factors that regulates the expression of VEGF, which plays an important role in angiogenesis in gliomas [154].

IDH1-related low-grade and anaplastic gliomas were not associated with an activation of HIF-1α and HIF-1α overexpression was restricted to necrotic areas [137,138,139]. IDH1 mutations and 2HG production were shown to inhibit prolyl-hydroxylase (PHD) enzymes, which inhibit HIF-1a, the major pro-angiogenic and pro-glycolysis transcription factor [155,156].

## 9. Aerobic Glycolysis and WNT/β-catenin Pathway in Glioma

Glucose is the major source of energy for mammalian cells. Glucose is metabolized to produce ATP (energy), through cytosolic glycolysis and oxygen-dependent mitochondrial metabolism. The entry of glucose into the TCA cycle is controlled by PDH (pyruvate dehydrogenase complex). Inhibition of PDH inactivates mitochondrial activity in gliomas [157]. A large part of the glucose supply is fermented to lactate; this phenomenon is called aerobic glycolysis or the Warburg effect [158].

Growing evidence reveals that all cancers, regardless of tissue or cellular origin, are a disease of impaired cellular energy metabolism [159]. In addition to the previously well recognized hallmarks of cancers [160,161,162], aerobic glycolysis is also a robust metabolic feature of most tumors [160,163,164]. Recent studies on gliomas in experimental models show the dependence of glioma cells on glycolysis as the primary source of energy [165]. Glioma cells are dependent on glycolysis for their primary source of energy [165], with upregulation of glycolytic metabolism [166,167]. Cancer cells can use aerobic glycolysis at all oxygen levels (i.e., normoxia, hypoxia, or hyperbaric oxygen) [168].

## 10. Normoxia and WNT/β-catenin Pathway Upregulation in Glioma

**Figure 2:**
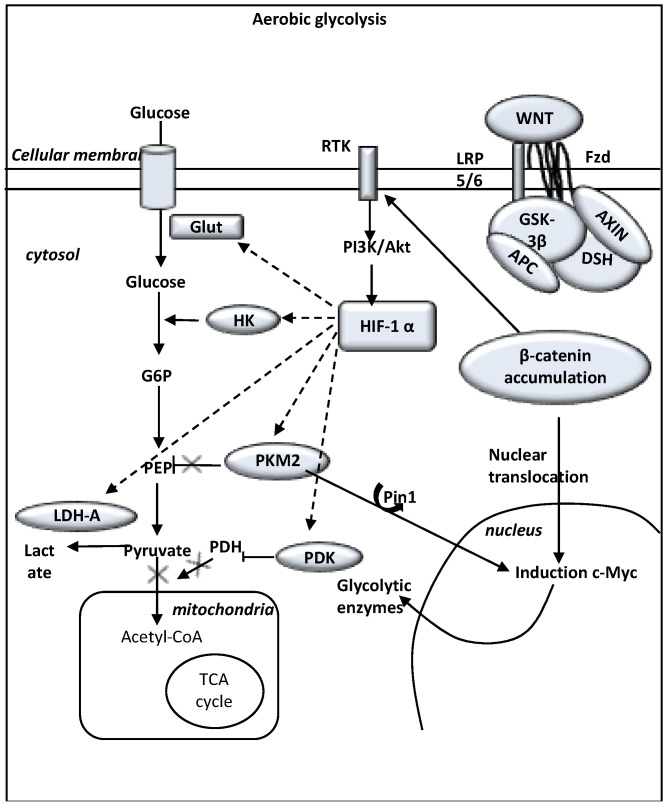
The WNT/β-catenin pathway and the Warburg effect (i.e., aerobic glycolysis). WNT ligands bind the complex Frizzled/LRP 5/6 receptors leading to LRP phosphorylation of the AXIN/APC/GSK-3β complex. β-catenin phosphorylation is inhibited and this prevents its degradation in the proteasome. β-catenin accumulates into the cytosol and then translocates to the nucleus to bind TCF-LEF co transcription factors. WNT-response gene transcription is stimulated (PDK, c-Myc, cyclin D, MCT-1). MCT-1 promotes the release of lactate out of the cell. WNT/β-catenin pathway activates tyrosine kinase receptors (TKRs). Activation of PI3K/Akt increases glucose metabolism. Akt-transformed cells induce HIF-1α stabilization, which largely diminishes the glucose entry into the TCA cycle. Stimulated HIF-1α activity increases expression of glycolytic enzymes (GLUT, HK, PKM2, LDH-A). Elevated aerobic glycolysis is observed with increased production of lactate and decreased mitochondrial respiration. HIF-1α induced PDK phosphorylates PDH, which results in cytosolic pyruvate being shunted into lactate through induction of LDH-A. PDK inhibits the PDH complex into mitochondria. Thus, pyruvate cannot be fully converted into acetyl-CoA and enter the TCA cycle. c-Myc and cyclin D also activate LDH-A, which converts cytosolic pyruvate into lactate. Activated PKM2 translocates to the nucleus through Pin1, then binds β-catenin and induces c-Myc expression. This activates GLUT, PKM2 and LDH-A in a positive feedback.

In cancer cells, early overexpression of the WNT/β-catenin pathway leads to induce aerobic glycolysis [169] (Figure 2).

In addition, WNT target genes, c-Myc and cyclin D1, also activate aerobic glycolysis [170]. c-Myc and PI3K/Akt signaling overexpression induces HIF-1α activation, which suppresses glucose entry into the TCA cycle [171,172]. Activation of canonical WNT signaling directly acts on aerobic glycolysis [173] and increases vessel development via the WNT target genes [174]. Activation of the canonical WNT/β-catenin pathway suppresses oxidative metabolism into the TCA cycle and promotes cell proliferation [174]. The WNT pathway induces the transcription of genes implicated in cell proliferation, c-Myc (through glutaminolysis, nucleotide synthesis and lactate dehydrogenase-A (LDH-A) activation) and cyclin-D1 (through G1) [170,175,176,177,178,179]. The WNT target gene c-Myc drives aerobic glycolysis and glutaminolysis [171,177]. c-Myc induces glutamine uptake into the cell and the mitochondria and favors aspartate synthesis [170].

WNT/β-catenin pathway stimulates RTK (such as PI3K signaling) activation in gliomas [180]. Downregulation of β-catenin reduces the expression of EGFR, Akt signaling and their downstream genes [181,182,183,184]. Aerobic glycolysis also occurs via PI3K/Akt signaling in cancer cells, glucose is taken up in excess and rewired towards protein and lipid synthesis [185,186,187], which induces cellular growth. Hyper activation of PI3K/Akt pathway is associated with an increased rate of glucose metabolism [188]. Under normoxic conditions, activation of the qPI3K/Akt pathway results in HIF-1α stimulation [189].

Phosphorylated STAT3 by IL-6 (interleukin 6) or LIF (leukemia inhibitory factor) is associated with overexpression of HIF-1α under normoxic conditions [190]. Β-catenin/TCF4 complex directly binds to the STAT3 gene promoter [191]. STAT pathways participate in tumorigenesis through up-regulation of genes encoding cell-cycle regulators (cyclin D1, c-Myc) and induces activation of VEGF [192].

It has been reported that HIF-1α can be activated by numerous oncogenes even under normoxic conditions (independent of hypoxia) [186,193,194,195]. HIF-1α is induced transcriptionally by PI3K/mTOR even under normoxic conditions through 4E-BP1 and STAT3 [190,196,197,198,199,200]. PI3K activates mTORC1 through Akt which inhibits by phosphorylation the tuberous sclerosis complex (TSC) [201,202]. mTOR-mediated HIF-1α induction mimics the effect of hypoxia and then leads to glycolysis [189]. Several genetic mutations (such as mutated genes encoding PDGFR, EGFR, p53, and PTEN) result in HIF-1α stabilization resulting in increased angiogenesis by upregulation of angiogenic factors [203,204,205,206,207]. HIF-1α activated initiates the transcription of target genes including glucose transporters, the MCT-4 and key pro-angiogenic effectors such as VEGF [149,208]. HIF-1α also promotes both glycolytic energy metabolism and angiogenesis and participates in the poor cancer prognosis [209,210].

c-Myc overexpression can also enhance LDH-A expression by promoting HIF-1α stabilization under normoxic conditions [211]. c-Myc cooperates with HIF-1α in activating several genes that encode glycolytic proteins, such as LDH-A [212]. LDH-A activation is associated with activation of the VEGF pathway [213,214,215,216].

Cancers cells use LDH-A to elevate the rate of glycolysis, ATP and lactate production [217]. HIF-1α and c-Myc are the major transcription factors of LDH-A [218,219,220,221]. Overexpression of HIF-1α is associated with LDH-A overexpression and poor survival in many cancers [215,222]. LDH-A activation is associated with activation of the VEGF pathway [213,214,215,216]. A high level of cytoplasmic LDH-A is correlated with cytoplasmic VEGF expression [215]. This association could be partly due to transcription factors HIF-1α and c-Myc [211]. Lactate plays an important role in angiogenesis, by stimulation of VEGF [223,224,225]. In gliomas, lactate also triggers HIF-1α activation in a hypoxia-independent manner by inhibition of HIF-1α proline hydroxylation [226]. Lactate stimulates HIF-1α activity in normoxic endothelial cells resulting in an increase of pro-angiogenic targets such as VEGFR2 [227]. VEGFR2 is the main transducer of pro-angiogenic effects of VEGF [228]. Lactate that arose from aerobic glycolysis induces the VEGF/VEGFR2 signaling pathway in a hypoxia-independent manner [229]. Acute acidic extracellular pH, which can be caused by elevated lactate production, was shown to promote upregulation of IL-8 and VEGF independently of hypoxia [230,231,232,233,234,235]. Lactate overexpression promotes angiogenesis [236] and increased extracellular lactate concentrations activates the VEGF pathway [237]. VEGF expression in glioma cells is independently regulated by pH and tissue pO2 [231]. Hypoxia and acidic pH have no synergistic effect on VEGF transcription [231].

## 11. Conclusions

**Figure 3:**
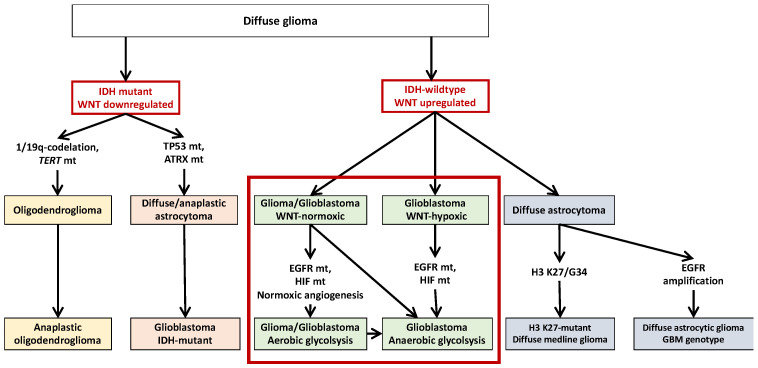
Features of diffuse glioma. In the 2016 WHO classification, diffuse astrocytic tumors and oligodendrogliomas can be defined by mutually exclusive genotypes (astrocytic, TP53 mt/ATRX mt; oligodendroglial, 1p/19q-codel/TERT mt) under the shared IDH mutation. IDH-wildtype tumors represent the main malignant glioblastoma or diffuse glioma genetically corresponding to glioblastoma. Our hypothesis is to add to this classification the notion of WNT pathway expression. IDH-mutant presents downregulation of the WNT pathway whereas IDH-wild-type presents upregulation of the WNT pathway. WNT pathway stimulation in the early stages of the tumor process is associated with normoxic glioma development with early angiogenesis process.

The majority of IDH wild-type glioblastomas refer to primary glioblastomas which occur in elderly patients and develop de novo. IDH-wild-type glioblastomas usually present short clinical history, without a pre-existing lower-grade precursor lesion. They are characterized by the upregulation of the WNT/β-catenin pathway showing an early tumor development under normoxic conditions for the main part [129]. In contrast, IDH mutant glioblastomas are typically observed in young adults and include the majority of secondary glioblastomas developed by the progression from a pre-existing diffuse or anaplastic astrocytoma [238]. The distinction of “IDH wild-type/WNT upregulated” and “IDH mutant/WNT downregulated” glioblastomas is major as these differences are not only biologically distinct subtypes but also associated with different clinical presentations, such as age and survival rate, with significantly shorter overall survival observed in patients with “IDH wild-type/WNT upregulated” glioblastoma [28]. Thus, opposed WNT/β-catenin pathway expression is observed between IDH wild-type and IDH mutant glioblastomas and this could be included in gliomas classification (Figure 3).

## Figures and Tables

**Table 1:** Opposed interplay between the WNT pathway and IDH1 mutations and the involved molecular targets.

Marker	Cell Pathways	Regulation	Subtypes	Prognosis	References
IDH1 mutations	NADPH-dependent reduction of alpha-ketoglutarate	HIF-1α reduction activity	Glioblastoma	Good survival rate	[136]
IDH1 mutations	TP 53 and total 1p/19q deletions	-	Oligodendroglia tumors	Good survival rate	[26]
IDH1-R132H mutation	β-catenin, TCF4 and LEF1 downregulation	Decrease WNT signaling	Glioma cells	Lower invasion	[126,134]
IDH1-R132H mutation	PI3K/Akt pathway decrease	Decrease WNT signaling	Glioma cells	Good survival rate	[131,132,133]
IDH1 mutations	HIF-1α reduction activity	Decrease WNT signaling	Restricted to necrotic areas	Poor survival rate	[137,138,139]
EGFR	β-catenin accumulation	Increase WNT signaling	Astrocytomas	Glioma progression and invasion	[98,99,101,102,103,104,105]
sFRP1 depletion	β-catenin accumulation	Increase WNT signaling	Glioblastoma	Inhibit Motility and Promote Growth	[112,113]
RTKs	WNT signaling	IDH1 decrease	Glioblastoma		[45]

## Supplementary Materials

The following are available online at https://www.mdpi.com/article/10.3390/biomedicines9060619/s1.
