# Supplementary material for: Opposed Interplay between IDH1 Mutations and the WNT/β-Catenin Pathway: Added Information for Glioma Classification
Source: Biomedicines. 2021 May 30;9(6):619. doi: 10.3390/biomedicines9060619 (PMC8229353; doi:10.3390/biomedicines9060619)
Supplement: Supplementary file 1 [file biomedicines-09-00619-s001.zip › biomedicines-1228711-supplementary.pdf]

Supplementary table: **Most commonly associated genetic (1), epigenetic (2), chromosomal (3) and molecular (4) aberrations in glioma entity**

**Glioma entity**	**Most commonly associated genetic (1), epigenetic (2), chromosomal (3) and molecular (4) aberrations**
Diffuse glioma	
Diffuse glioma, IDH mutant	(1) IDH1 or IDH2, TP53, ATRX mutation; (2) G-CIMP; (3) trisomy 7 or 7q gain, LOH 17p
Anaplastic astrocytoma, IDH mutant	(1) IDH1 or IDH2, TP53, ATRX mutation; (2) G-CIMP; (3) trisomy 7 or 7q gain, LOH 17p
Oligodendroglioma, IDH mutant and 1p/19q co-deleted	(1) IDH1 or IDH2, TERT, CIC, FUBP1 mutation; (2) G-CIMP; (3) 1p and 19q co-deletion
Anaplastic oligodendroglioma, IDH mutant and 1p/19q co-deleted	(1) IDH1 or IDH2, TERT, CIC, FUBP1, TCF12 mutation; (2) G-CIMP; (3) 1p and 19q co-deletion
Glioblastoma, IDH mutant	(1) IDH1 or IDH2, TP53, ATRX mutation, homozyguous CDKN2A/p14^{ARF} deletion; (2) G-CIMP; (3) trisomy 7 or 7q gain, LOH 17p, 10q deletion ; (4) G-CIMP, TP53 mutation, ATRX mutation, CDKN2A/B deletion.
Glioblastoma, IDH wild-type	(1) TERT, PTEN, TP53, PIK3CA, PIK3R1, NF1, H3F3A mutation, EGFR, PDGRA, MET, CDK4, CDK6, MDM2, MDM4 amplification, EGFRvIII deletion mutation; (2) MGMT promoter methylation; (3) trisomy 7 or 7q gain, monosomy 10, double minute chromosomes; (4) PDGFRA amplification, TERTp mutation, EGFR amplification, CDK4/CDK6 amplification, MDM2/MDM4 amplififcation, PTEN mutation,
Diffuse midline glioma, H3-K27M mutant	(1) H3F3A or HIST1H3B/C K27M, TP53, PPMD1, ACVR1, FGFR1 mutation, PDGFRA, MYC/MYCN, CDK4/6, CCND1-3, ID2, MET amplification; (2) global histone H3 hypomethylation
Pediatric low-grade diffuse glioma	(1) MYB or MYBL rearrangement, FGFR1 duplication
Pilocytic astrocytoma	(1) BRAF or RAF1 fusion genes, BRAF-V600E, NF1, KRAS, FGFR1, PTPN11 mutation, NTRK2 fusion genes
Pleomorphic xanthoastrocytoma	(1) BRAF-V600E mutation, CDKN2A/p14^{ARF} homozygous deletion
Pleomorphic xanthoastrocytoma	(1) TSC1 or TSC2 mutation

Masui, K.; Mischel, P.S.; Reifenberger, G. Molecular Classification of Gliomas. *Handb. Clin. Neurol.* **2016**, *134*, 97–120, doi:10.1016/B978-0-12-802997-8.00006-2.
